# Oral administration of wild plant‐derived minerals and red ginseng ameliorates insulin resistance in fish through different pathways

**DOI:** 10.14814/phy2.15667

**Published:** 2023-04-20

**Authors:** Kiyomi Takase, Izuru Kakuta

**Affiliations:** ^1^ Research Center for Creative Partnerships Ishinomaki Senshu University Ishinomaki 986‐8580 Japan; ^2^ Faculty of Science and Engineering Ishinomaki Senshu Univerisity Ishinomaki 986‐8580 Japan

**Keywords:** AMPK signaling pathway, fish, insulin resistance, PI3K/Akt pathway

## Abstract

Many kinds of fish are characterized by a limited efficiency to use carbohydrates. For this reason, raw fish and mixed feed containing a lot of fish meal have been used as feed for fish farming. However, continuing to use high‐protein diets not only increases the cost of fish farming, but may also fuel animal protein shortages. Furthermore, carbohydrates are added to improve the texture of the feed and act as a binding agent and are usually contained at 20% in the feed. It makes sense, therefore, to find ways to make good use of carbohydrates rather than wasting them. The physiological mechanisms of glucose intolerance in fish are not yet well understood. Therefore, we investigated the glucose utilization of fish, omnivorous goldfish *Carassius auratus* and carnivorous rainbow trout *Oncorhynchus mykiss*. Furthermore, the effects of oral administration of wild plant‐derived minerals and red ginseng on the glucose utilization in these fish muscle cells were investigated. As a result, we found the following. (1) An extremely high insulin resistance in fish muscle and the symptom was more pronounced in carnivorous rainbow trout. (2) Administration of wild plant‐derived minerals promotes the translocation of the insulin‐responsive glucose transporter GLUT4 to the cell surface of white muscle via activation of the PI3 kinase axis, whereas administration of red ginseng not only promotes GLUT4 transfer and translocation to the cell surface of white muscle via AMPK activation as well as promoting glucose uptake into muscle cells via a pathway separate from the insulin signaling system. (3) In fish, at least goldfish and rainbow trout, both PI3K/Akt and AMPK signaling cascades exist to promote glucose uptake into muscle cells, as in mammals.

## Graphical abstract

Fish has a very low utilization rate of carbohydrate (glucose) for the high insulin resistance.But, administration of wild plant‐derived minerals promotes the translocation of the insulin‐responsive glucose transporter GLUT4 to the cell surface of white muscle via activation of the PI3 kinase axis, and administration of red ginseng not only promotes GLUT4 transfer and translocation to the cell surface of white muscle via AMPK activation as well as promoting glucose uptake into muscle cells via a pathway separate from the insulin signaling system.

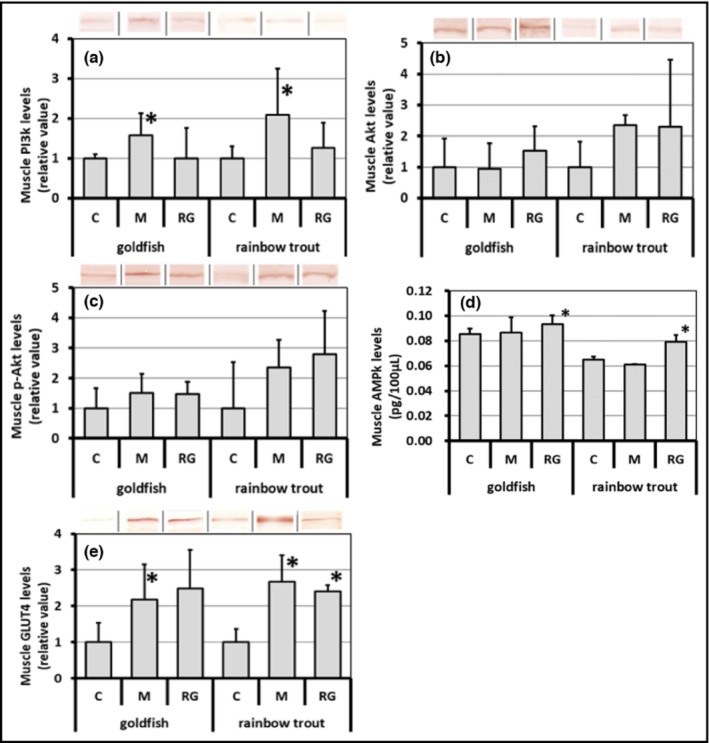

## 1 INTRODUCTION

Many cultured fish are carnivorous, and supply of high‐protein feed is essential (Ogino, 1980). In recent years, however, the cost of aquaculture has continues to rise as the prices of small fish and fishmeal have risen due to a decrease in the amount of global resources and the increase in the number of farmed fish to cope with food shortages. Therefore, in addition to the use of plant‐based ingredients such as soybean meal and corn gluten meal as substitutes for miscellaneous small fish and fishmeal, and animal‐based ingredients such as feather meal and chicken meal (use of low‐fishmeal feed), it is important to increase the utilization of lipids and carbohydrates, which are inexpensive and can be supplied stably, as substitutes for protein raw materials.

The utilization of lipids is progressing (Furuichi, 1983). However, the use of carbohydrates in feed is not necessarily progressing. The amount of carbohydrates in commercial fish feed is approximate 40% for carp and goldfish, about 25% for trout, and around 15% for young yellowtail and flounder, which are not low figures (Shimeno, 1980). It is thought that the reason why carbohydrate utilization in the diet is not progressing is that most fish have low carbohydrate utilization capacity, and exhibit glucose intolerance, in which hyperglycemia persists for a long time after carbohydrate intake (Legate et al., 2001; Polakof et al., 2012). In addition, no small amount of carbohydrate is added to improve the texture of the feed and act as a binding agent. So it makes sense to find ways to put carbs good use, rather than wasting them.

In mammals, insulin‐binding to the receptors on the cell membrane induces that the translocation of GLUT4 vesicles, the docking and fusion with the plasma membrane, as a result that blood glucose takes into the cells (Lauritzen & Schertzer, 2010; Wallberg‐Henriksson & Zierath, 2001). The phosphatidylinositol 3‐kinase (PI3K)/Akt pathway is the most important of these (Dong et al., 2013; Thiel et al., 2021). In addition, it has been reported that activation of AMP‐activated protein kinase (AMPK) also enhances the regulation (transcription and translation) of the insulin‐responsive glucose transporter GLUT4, thereby supporting glucose uptake by insulin (Kahn et al., 2005; Shrestha et al., 2021; Zhao et al., 2018).

By the way, AMPK activation positively regulates signaling pathways that replenish the cellular ATP supply, such as fatty acid oxidation and autophagy. On the contrary, it negatively regulates ATP‐consuming biosynthetic processes such as gluconeogenesis, lipid, and protein synthesis. In this reaction, the activation of AMP‐activated protein kinase (AMPK) leads to decrease in plasma glucose concentration. That is, an insulin‐independent glucose transport pathway, an AMPK signaling pathway, for the elevation of the uptake of glucose from blood to cells is also existence (Egawa et al., 2009; Kishi et al., 1998; Magnone et al., 2020a).

It was reported that the AMPK pathway involved in glucose metabolism was present in brown trout muscle cells (Magnoni et al., 2012), whereas in tilapia the AMPK pathway, which facilitates glucose uptake into muscle cells, was reported to be absent (Guan et al., 2021). Therefore, the physiological mechanisms underlying glucose intolerance in fish, including the presence of the AMPK pathway, are still poorly understood.

We reported previously that the 2DG uptake rate in the lateral white muscle tissue from goldfish administrated orally wild plant‐derived minerals and red ginseng improved markedly the insulin sensitivity in trunk muscle (lateral white muscle) of goldfish (Takase & Kakuta, 2020). Minerals derived from wild plants, which consist of tree ashes, wild grasses, and seaweed, have high antioxidant capacity and improved insulin resistance in type 2 diabetes model mice (Takase & Kakuta, 2017). Red ginseng is a crude drug used to improve type II diabetes in mammals (Choi et al., 2013; Saito et al., 2011). However, the above report did not examine the mechanism in fish. In this study, therefore, the existence and contribution of PI3/Akt and AMPK signaling cascades, which are involved in glucose uptake in muscle cells, the mechanism that improves insulin resistance in lateral white muscle cells from two types of fish, goldfish, an omnivorous fish and rainbow trout, a carnivorous fish, administered orally wild plant‐derived minerals or red ginseng were investigated.

## 2 MATERIALS AND METHODS

### 2.1 Ethics statement

This study was conducted at Department of Biological Science, Ishinomaki Senshu University, Japan. All experimental protocols and methods were conducted in accordance with “Raising laboratory animals Standards for storage and pain reduction” by the Ministry of the Environment of Japan.

### 2.2 Fish and bio‐materials administration study

Goldfish *Carassius auratus* (about 15 g body weight) which is an omnivorous fish and rainbow trout *Oncorhynchus mykiss* (about 100 g body weight) which is a carnivorous fish were used in this study. The goldfish and rainbow trout were held in a circulating filtration tank adjusted to 20°C and 13°C, respectively. After preliminary rearing for more than 1 week, they were divided into three groups of 20 fish each and started a bio‐materials feeding test.

The control group was reared on a commercial diet, and the other two groups fed the same diet containing wild plant‐derived minerals (Group M; 100 mg/kgBW/day; Yatsuka Co., Ltd.) or red ginseng (Group RG;100 mg/kgBW/day) for 3 weeks. Each group was fed for 5 days per week. The commercial feed and test diets were administered at a daily dose of 1% of body weight for goldfish and 2% of the body weight of rainbow trout. After 3 weeks, the fish were taken up and the following indices were investigated.

The proximate composition of the commercial diets for goldfish and rainbow trout used in this experiment was follows (%): includes crude protein; 36.8 and 46.0, crude lipid; 4.0 and 10.0, crude ash; 8.2 and 15.0, carbohydrate fibers; 3.0 and 3.0. Elemental composition of wild plant‐derived minerals administered to fish in this experiment was shown in Table 1.

**TABLE 1:** Elemental composition of wild plant‐derived minerals administered to fish in this experiment.

Element	Content (g/100 g)
Calcium	1 9.5
Potassium	1 2.8
Sodium	3.0
Magnesium	2.6
Phosphorus	1.7
Iron	0.39
Manganese	0.32
Sulfur	0.080
Iodine	0.043
Zinc	0.028
Chromium^a^	0.021
Fluorine	0.015
Boron	0.0085
Lead	0.0066
Copper	0.0065
Vanadium	0.0040
Nickel	0.0015
Arsenic	0.0014
Molybdeum	0.0007
Cobalt	0.0001
Selenium	0.000018

^a^ Not containing hexavalent chromium.

### 2.3 Intraperitoneal glucose tolerance test

Glucose tolerance tests were performed 3 weeks after the start of the culture administered test materials. After the intraperitoneal administration of glucose (1500 mg/kg body weight) to fish, blood was collected with the passage of time from the tail vessels of fish using heparin sodium treated syringes. MS‐222 (solution concentration at 100 mg/L) was used to anesthetize the sample fish. The plasma was divided by centrifugation at 800 *g* for 10 min at room temperature, and the glucose concentration (glucose CII test wako, Wako Pure Chemical Industries Co., Ltd.) in plasma was measured.

### 2.4 Measurement of 2‐deoxy‐D‐glucose (2DG) uptake rate in isolated lateral white muscle

Three weeks after the start of fish culture test, four fish without intraperitoneal glucose injection from each group were used for this experiment. After blood sampling, the lateral white muscle of the body was taken from the base of the anterior side of the dorsal fin of the trunk, aligned to the same shape and size (about 5 mm × 5 mm × 3 mm), and insulin resistance of the muscle stripes was estimated by measuring the uptake amount of 2‐deoxy‐D‐glucose (2‐deoxy‐D‐[1,2‐3H(N)] glucose: 2DG, Wako Pure Chemical Industries, Co.).

A 2DG uptake test into white muscle samples was carried out according to the method of Ogihara et al. (2001) as follows: The muscles were initially preincubated for 60 min at 37°C in oxygenated Krebs‐Ringer‐phosphate‐Hepes buffer (KRPH buffer, pH 7.5) containing 8 mM glucose, 32 mM mannitol, and 0.1% bovine serum albumin. Muscles were then incubated for 20 min in an identical medium in the absence or presence of human insulin (Wako Pure Chemical Industries, Co.) at 0.25, 0.5, 1.0, 1.5, and 2.0 mIU/mL. Subsequently, the muscle samples were washed with KRPH buffer supplemented with 40 mM mannitol and 0.1% BSA for 10 min, followed by 1.5 mL of KRPH buffer supplemented with 8 mM 2DG, 32 mM mannitol, 2 mM sodium pyruvate, and 0.1% BSA. After incubating in KRPH buffer for 20 min to uptake 2DG, the reaction was stopped by washing three times with PBS containing 200 μM phloretin (Sigma‐Aldrich). Cells were then harvested into microtest tubes using 1 mL of 10 mM Tris–HCl buffer (pH 8.0) per sample and stored at −80°C until 2DG amount determination.

After the procedure for taking 2DG, trypsin was added to the thawed sample to a final concentration of 0.5% and allowed to react (lysing) at 37°C for 4 h. Heating treatment of lysed samples at 80°C for 20 min, followed by centrifugation at 15,000 *g* for 20 min. The supernatant obtained was used as a sample for 2DG quantification. The amount of 2DG taken up into cells was measured by the method of Saito et al. (2011).

### 2.5 Quantification of GLUT4 and GLUT4 transportation‐related factors

The translocation‐related factors of GLUT4 in muscle, PI3 kinase, Akt, phosphorylated Akt, and GLUT4, were measured by western blotting. In other words, 3 weeks after the administration of wild plant‐derived minerals and red ginseng, protein solutions were prepared from the muscle tissue collected from the lateral white muscle, separated by SDS‐PAGE, and the target proteins were detected by western blot. In order to detect carriers translocated onto the cell membrane in GLUT4, the cell membrane fraction was recovered and used for experiments according to the method of Nishiumi et al. (Nishiumi & Ashida, 2007). Obtained bands were measured by image analysis software ImageJ. Glucose transporter GLUT4 antibody (NOVUS Biological Co.; NBP1‐49533), anti‐PI 3‐kinase, p85 (Sigma‐Aldrich Co.; ABS1856), antibody Akt (CST Japan Co., Ltd.; #9272), and antibody phosphor‐Akt Ser 473 (CST Japan Co., Ltd.; #9271) were used as the primary antibodies, as previously reported (Takase & Kakuta, 2017), and HRP‐linked anti‐rabbit IgG (Sigma‐Aldrich Co.; A0545) was used as a secondary antibody. The mass of each molecule is as follows: PI 3 kinase 83.5 kda, Akt 56 kda, phosphorylated Akt 60 kda, and GLUT4 30 kda. AMP kinase activity was measured by ELISA using an CycLex AMP kinase assay kit (MBL Life science Co.).

### 2.6 Statistical analysis

The numbers in the text are expressed as mean ± standard deviation (*n* = 4) unless otherwise stated. The sample size for comparing the mean values between the two groups was calculated using the free software EZR under the following conditions: Type I error; 0.05, power; 0.08, sample size ratio; 1:1. Tukey's multiple range test was used for multiple comparisons based on one‐way ANOVA, with *p* < 0.05 as the limit of significance.

## 3 RESULTS

During the culture period, experimental fish in each group ate well, and no abnormality was observed in behavior or appearance.

**FIGURE 1:**
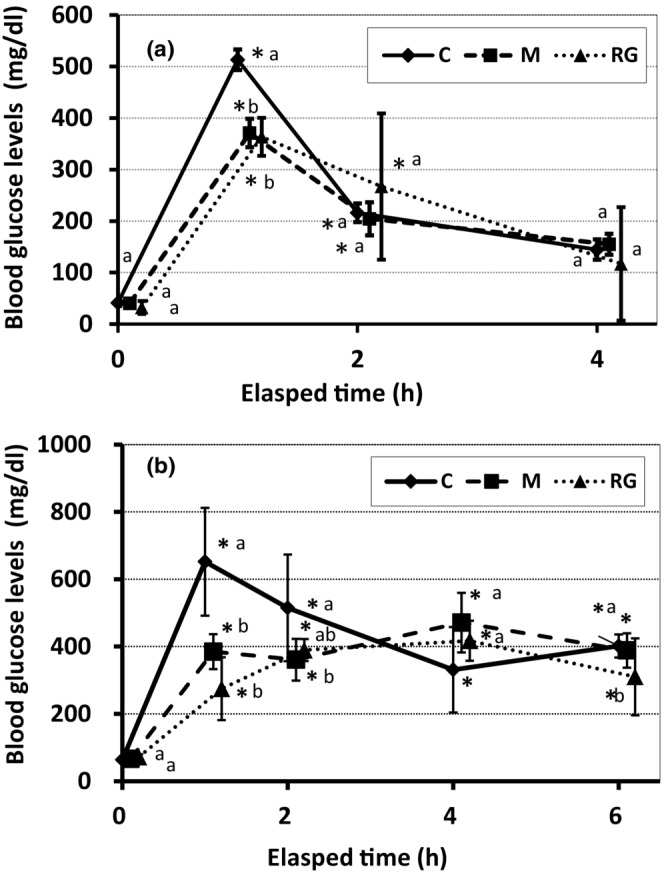
Intraperitoneal glucose tolerance test (IPGTT) and plasma glucose levels of goldfish (a) and rainbow trout (b) administered orally bio‐resource agents such as a mineral mixture derived from wild plants and red ginseng. C, control group; M, wild plant‐derived minerals group (100 mg/kgBW/d), RG, Red ginseng group (100 mg/kgBW/d) Bio‐resources were administered orally for 3 weeks. Fish were administered intraperitoneally glucose at the dose of 1500 mg/kg BW. Data are shown as mean ± SD, *n* = 4. *Significant difference from the initial (*p* < 0.05). a,b: Different letters indicate significant differences among groups (*p* < 0.05).

Figure 1 shows the effects of orally administered wild plant‐derived minerals and red ginseng on plasma glucose levels of goldfish (a) and rainbow trout (b) in an intraperitoneal glucose tolerance test. In goldfish, the plasma glucose concentration in the control group reached the highest value of about 10 times of the preload value after 1 h the challenge, then decreased remarkably, and 4 h after the glucose loading it reached almost the initial levels. In groups M and RG, the highest values were shown in the same manner as the control group 1 h after the glucose loading, but the values in both groups were about 20% lower than those of the control group. Two hours after the load, these were reaching almost the same level as the control group and decreased to the initial levels after 4 h. In rainbow trout, the plasma glucose concentration in the control group reached the highest value of around 10 times the preload value at 1 h after the glucose loading, and then gradually decreased. On hour 6, however, the value was still significant higher, which was several times of the preload value. In M and RG groups, the change in blood glucose level after the glucose load was gradual, no significant peak was observed in the first hour after the load, and almost the same high level was observed from 1 h to 6 h after the load. At 2 h after glucose loading, the M group showed a significant lower value. Six hours after glucose loading, only group RG showed significantly lower values than the control.

**FIGURE 2:**
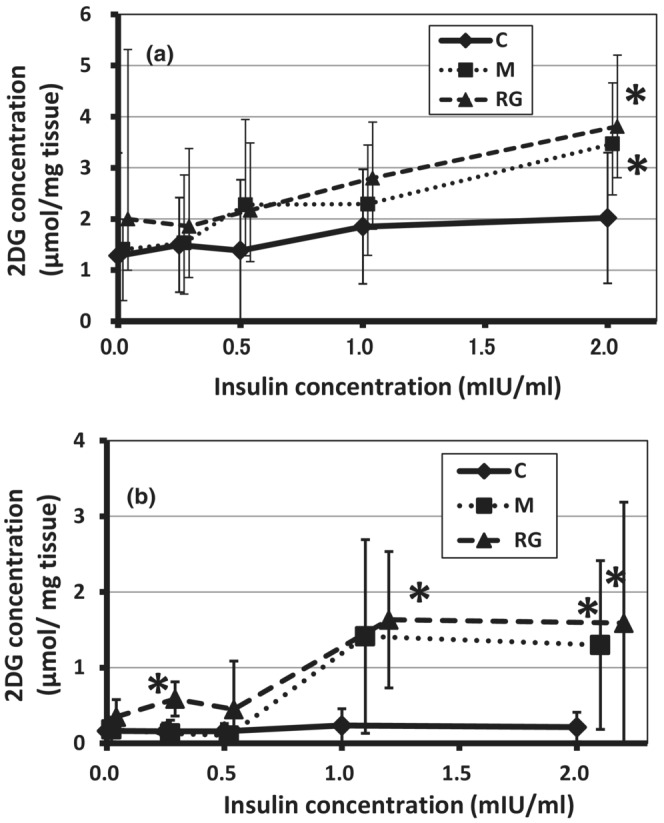
Changes in the 2DG uptake in the absence or the presence of insulin of trunk muscle tissue from goldfish (a) and rainbow trout (b) administered orally bio‐resource agents such as mineral mixture derived from wild plants and red ginseng. C, control group; M, wild plant‐derived minerals group (100 mg/kgBW/d); RG, red ginseng group (100 mg/kgBW/d) Bio‐resources were administered orally for 3 weeks. Data are shown as mean ± SD, *n* = 4. *Significant difference from the control (*p* < 0.05).

Figure 2 shows the effects of oral administration of wild plant‐derived minerals and red ginseng on the rate of 2DG uptake in the lateral white muscle of goldfish (a) and rainbow trout (b) with the absence and presence of insulin. In the absence of insulin, there was no significant difference in the rate of 2DG uptake in isolated lateral white muscle of both fish species between the test (M and RG) groups and the control group (goldfish; 2.0 μmol/mg tissue, rainbow trout; 0.2 μmol/mg tissue). However, in RG group, the values in the absence of insulin for both goldfish and rainbow trout increased to about 1.5 times those of the control group. The latter also showed a significant increase in insulin concentration at an extremely low insulin concentration of 0.25 mIU/mL. In the presence of insulin (2 mIU/mL), the uptake rate of 2DG in goldfish in both M and RG groups increased to about twice that of the control, showing significantly higher values. In rainbow trout, in the presence of insulin (1 and 2 mIU/mL), the 2DG uptake rate in the lateral white muscle was significantly higher in both groups, 5–10 times higher than in the control group.

**FIGURE 3:**
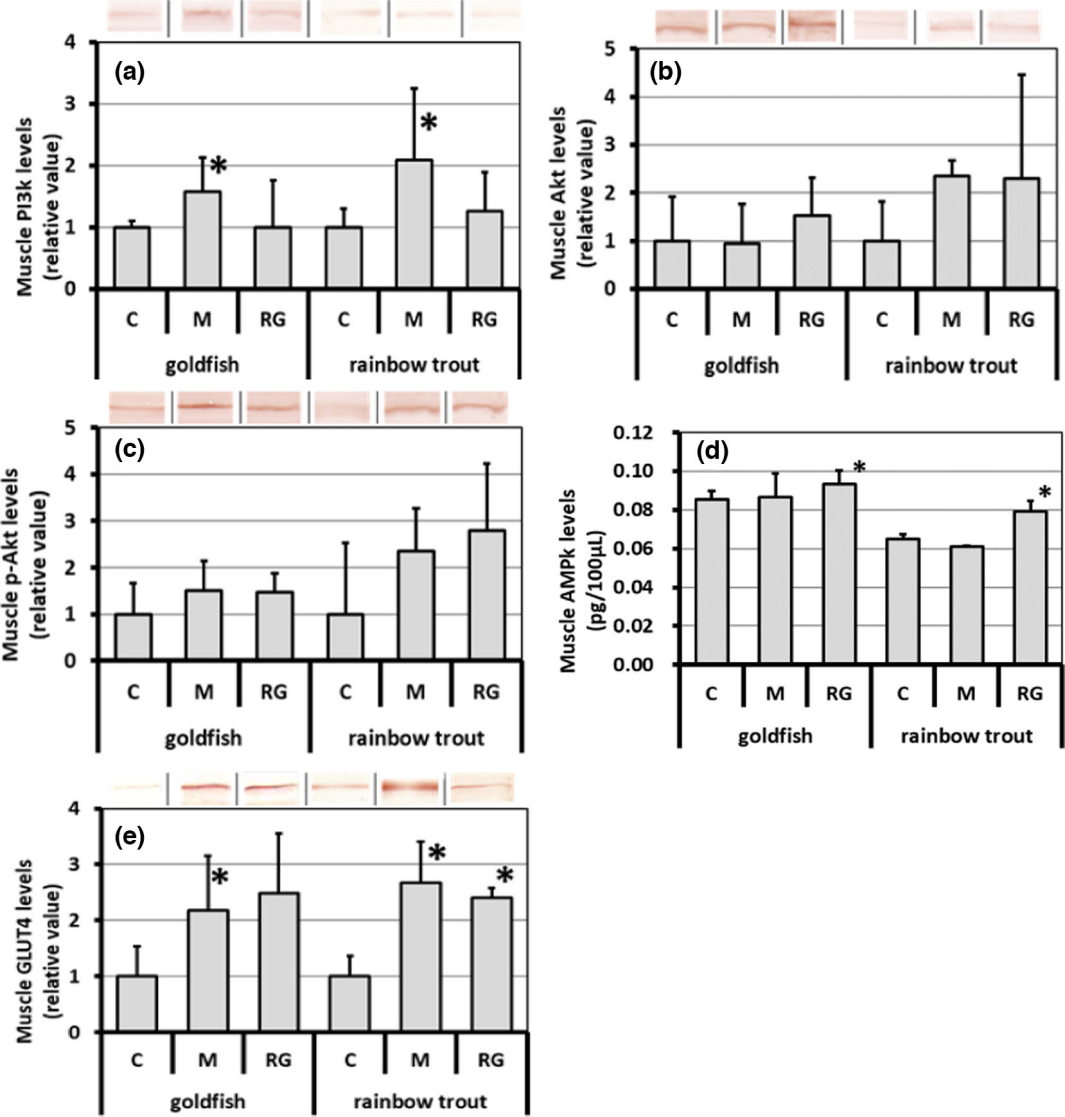
Changes in the GLUT4 troncelocation relative factor and GLUT4 in muscle tissue from goldfish and rainbow trout administered orally bio‐resource agents such as wild plant‐derived minerals and red ginseng. C, control group; M, wild plant‐derived minerals group (100 mg/kgBW/d); RG, red ginseng group (100 mg/kgBW/d) (a); PI3 kinase, (b); Akt, (c); phosphate Akt, (d); AMP kinase, (e); GLUT4 located on the membrane surface of muscle cells. Bio‐resources were administered orally for 3 weeks. Data are shown as mean ± SD, *n* = 4. *Significant difference from the control (*p* < 0.05).

Figure 3 shows the effects of administration of wild plant‐derived minerals and red ginseng on GLUT4 translocation‐related factors and GLUT4 levels in goldfish and rainbow trout. When wild plant‐derived minerals were administered, the levels of PI3 kinase (a) and GLUT4 located on the muscle cell surface (e) were remarkably elevated in both species, in particular the latter was more than twice as high as in the control group. On the contrary, when red ginseng was administered, the levels of AMP kinase (d) and GLUT4 were significantly increased in both fish species. In rainbow trout, in particular, the former was about 1.3 times and the latter being more than twice as high as in the control group. There were no statistically significant changes in the levels of Akt (b) and phosphorylated Akt (c) in either group compared with the control group. In rainbow trout, however, both indices tended to be higher after the administration of wild plant‐derived minerals and red ginseng (each mean value increased to more than twice as high as the control).

## 4 DISCUSSION

Mammals have both PI3K/Akt and AMPK signaling cascades, which are essential for glucose homeostasis (Kanai et al., 1993; Magnone et al., 2020; O'Neill, 2013; Zhou et al., 2017). The former works in the presence of insulin (Kanai et al., 1993), the latter in the presence and absence (Magnone et al., 2020b; Zhou et al., 2017) of insulin. On the contrary, in tilapia (Guan et al., 2021), orally administered rosiglitazone has been shown to upregulate her IRS‐1, PI3K, and GLUT‐4 mRNA levels in skeletal muscle, increase insulin sensitivity and promote glucose uptake. However, the AMP kinase activator metformin has been also reported not only to be unable to improve glucose homeostasis in fish but also to counteract the effects of insulin, that is, elevate insulin resistance in fish muscle. This means that the AMPkinase signaling cascade is absent in fish at least.

Previous study showed that the 2DG uptake rate in the lateral white muscle from goldfish administered orally wild plant‐derived minerals and red ginseng improved markedly (Takase & Kakuta, 2020). However, it did not examine the mechanism. In the present study, therefore, we investigated the effects of wild plant‐derived minerals and red ginseng on the activity of PI3/Akt and AMPK pathways, which promote glucose uptake in white muscle from carnivorous rainbow trout as well as omnivorous goldfish.

First, as a glucose tolerance test (intraperitoneal load) on goldfish and rainbow trout, we performed a glucose load of approximately the same amount as in a glucose tolerance test conducted in mammals (intraperitoneal administering 1–2 g of glucose per kg of body weight, that is, dose roughly equivalent to 75 g oral glucose tolerance test in humans). Four hours after the glucose load, elevated plasma glucose levels almost returned to the initial levels in goldfish. In rainbow trout, the levels of plasma glucose after 6 h were maintained significantly higher than before glucose load. It is known that in mammals blood glucose levels returned to baseline levels within 2 h after glucose challenge. These changes in blood glucose levels indicates that fish have a lower glucose utilization capacity than mammals, and the capacity is extremely low in rainbow trout, which is a carnivorous fish. In fact, the uptake rate of 2DG in the lateral white muscle under an insulin concentration of 2 mIU/mL was 2 μmol/mg tissue from goldfish and 0.2 μmol/mg tissue from rainbow trout muscle was remarkably low, compared to mammalian values measured by using the biceps femoris muscle derived from mice (about 75 μmol/mg tissue; Takase & Kakuta, 2020). That is, (1) it was found that an extremely high insulin resistance in fish muscle and the symptom was more pronounced in carnivorous rainbow trout.

Second, oral administration of wild plant‐derived minerals and red ginseng contributed to the prevention and improvement of insulin resistance in white muscle through different mechanisms. Namely, (2) administration of wild plant‐derived minerals promotes the translocation of the insulin‐responsive glucose transporter GLUT4 to the cell surface of white muscle via activation of the PI3 kinase axis, whereas administration of red ginseng not only promotes GLUT4 transfer and translocation to the cell surface of white muscle via AMPK activation as well as promoting glucose uptake into muscle cells via a pathway separate from the insulin signaling system.

Third, (3) this study revealed that in fish, at least goldfish and rainbow trout, both PI3K/Akt and AMPK signaling cascades exist to promote glucose uptake into muscle cells, as in mammals.

Wild plant‐derived minerals used in this experiment are ash obtained by burning various natural wild plants such as tree leaves, wild grasses, and seaweeds in a reducing atmosphere. It is mainly composed of phosphorus, iron, zinc, copper, manganese, molybdenum, selenium, and sulfur and exhibits high reducibility. It has been reported that magnesium, zinc, chromium, and vanadium are involved in insulin resistance or have ameliorating effects (Cekić et al., 2011; Hopfner et al., 1999; Karmaker et al., 2009; Solati et al., 2014). Each daily intake (mg) of magnesium, zinc, chromium, and vanadium calculated from the inorganic element composition of the plant minerals used in this study is 2.6, 0.028, 0.021, and 0.004; however, these are extremely small compared to their effective amounts. It has also been reported that the intake of materials with high antioxidant capacity increases the tissue's ability to utilize glucose (lowers muscle insulin resistance; Hiromura & Sakurai, 2008; Ishiki et al., 2013). The abovementioned action of wild plant‐derived minerals is thought to have been brought about by their combination, but continuous investigation is necessary for the factors that produce the effect.

On the contrary, it has been reported that panax ginseng such as red ginseng is effective in improving type 2 diabetes by activating AMP kinase with the active ingredient ginsenosides (Choi et al., 2013; Saito et al., 2011). In this study, it was found that oral administration of red ginseng to fish showed the same glucose utilization‐enhancing effect as that to mammals. However, since red ginseng has a unique aroma, while there is no problem with adding red ginseng at the current level, if the amount added to the diets is increased, it may be necessary to pay attention to the odor.

The results of this study showed that administration of wild plant‐derived minerals and red ginseng was effective in improving glucose utilization in both omnivorous goldfish and carnivorous rainbow trout. In particular, the administration of these bio‐resource agents greatly promoted the glucose utilization of carnivorous fish. It is thought that it will provide an effective means for reducing the protein content in diets (producing low‐protein diets), that is, for suppressing wasteful use of protein resources and reducing aquaculture costs. However, at present, even if the administration of these bio‐resource agents increases the rate of glucose uptake in muscles, the rate of glucose uptake into muscles in fish is considerably lower than that in mammals (approximately 1/15–1/40). Therefore, first of all, we would like to proceed with efforts aimed at improving the effects by optimizing the dosage of minerals and red ginseng alone, the effect of combined administration, the administration period, the timing of administration, and so on.

Insulin secretion from pancreatic β‐cells is important for glucose uptake into muscle cells, but it has been reported that fish do not have as high insulin‐secreting ability as mammals. As there are reports that insulin secretion is promoted by administering arginine (Ince & Thorpe, 1977) and L‐alanine (Jubouri et al., 2021), the research to secure the amount of insulin released and to optimize the timing of insulin release is also essential in order to promote glucose utilization.

There are some reports that not only the activity of enzymes related to carbohydrate digestion function (Buddington & Hilton, 1987; Enes et al., 2009; Gilannejad et al., 2018; Polakof et al., 2012; Shimeno, 1980), but also the ability of glucose absorption in the intestine of fish are as well as those of mammals. However, no consistent results have been obtained regarding the intestinal absorption capacity of glucose in fish. In order to improve the utilization rate of carbohydrates contained in diets in a true sense, it is also necessary to increase the rate of glucose uptake in the digestive tract. The activating of SGLT, GLUT1, and Na‐K‐ATPase are required for this upregulation (SGLT for the former and of GLUT1 and Na‐K‐ATPase for the latter; Xu et al., 2022).

In order to promote the utilization of carbohydrates (including glucose) in the diet and disseminate the high‐carbohydrate and low‐protein diets for fish culture as soon as possible; in addition to efficient glucose absorption in the gastrointestinal tract, an appropriate supply of insulin from the pancreatic β‐cells and an efficient utilization of glucose absorbed in fish bodies must be achieved. Therefore, it is necessary to conduct a wide range of research on the enhancement and the adjustment of muscle and pancreatic functions related to glucose utilization in fish bodies, and promotion of glucose absorption in the intestine.

## FUNDING INFORMATION

No funding information provided.
